# Biomarkers in the Diagnosis of Aspergillosis

**DOI:** 10.3390/jof12040259

**Published:** 2026-04-03

**Authors:** Laura Rivera-Agudelo, Diego H. Cáceres, Julián C. Arango-Rincón, Julio C. Jaramillo-Alzate, Alejandra Zuluaga-Rodriguez, Álvaro L. Rúa-Giraldo

**Affiliations:** 1 Grupo de Micología Médica y Experimental, Corporación para Investigaciones Biológicas CIB-UdeA-UPB-UDES, Medellín 050010, Colombia; laura.riveraa@outlook.com (L.R.-A.); julian.arango@udea.edu.co (J.C.A.-R.); alvaro.rua@udea.edu.co (Á.L.R.-G.); 2 IMMY, 2701 Corporate Centre Dr, Norman, OK 73069, USA; 3 Center of Expertise in Mycology Radboudumc/CWZ, 6525GA Nijmegen, The Netherlands; 4 Studies in Translational Microbiology and Emerging Diseases (MICROS) Research Group, School of Medicine and Health Sciences, Universidad del Rosario, Bogota 111221, Colombia; 5 Escuela de Microbiología, Universidad de Antioquia, Medellín 50010, Colombia; 6 Grupo Inmunología celular e inmunogenética (GICIG), Universidad de Antioquia, Medellín 50010, Colombia; 7 Grupo de Microbiología Ambiental, Universidad de Antioquia, Medellín 50010, Colombia

**Keywords:** biomarkers, aspergillosis, *Aspergillus*, lung diseases, fungal, antigens

## Abstract

Aspergillosis is a group of different invasive and non-invasive diseases affecting the lung and other organs, caused by species of the genus *Aspergillus*. Additionally, complications associated with treatment and the increasing emergence of antifungal-resistant strains of *Aspergillus* spp. are high-impact problems. For these reasons, a combined approach of diagnostic tests is necessary to reach an accurate and timely diagnosis. This review aims to describe some biomarkers and their usefulness for the diagnosis of aspergillosis. Among the findings obtained in different studies, the nature, analytical performance and usefulness for diagnosis in different clinical forms of this disease are described. Currently, the main biomarkers used in the diagnosis of *Aspergillus* disease fall into three categories: membrane components, specific DNA sequences and metabolic products. The detection of biomarkers is one of the most important innovations of recent decades in the field of medical mycology, as it is a diagnostic aid that allows the timely detection of infections and decreases the time of administration of antifungal therapy.

## 1. Introduction

Infections caused by *Aspergillus* spp. are associated with high mortality in immunocompromised patients [1]. Although the epidemiology of fungal diseases has changed in recent decades, species of the genus *Aspergillus* remain among the principal fungi responsible for severe illnesses [2]. *Aspergillus* is a genus of ubiquitous, saprophytic filamentous fungi that primarily inhabit decaying plant material [3,4], although they have also been isolated from dust during hospital construction activities [5]. Currently, this genus is classified into 6 subgenera, 27 sections, and 75 series comprising 453 species [6,7]. However, *Aspergillus fumigatus*, *Aspergillus flavus*, *Aspergillus terreus*, *Aspergillus niger*, and *Aspergillus nidulans* are recognized as the most common human pathogens [3]. Species-level identification is highly relevant for selecting appropriate antifungal treatment for affected individuals [8].

In general, pulmonary fungal infections represent a high cost for healthcare due to the prolonged hospitalization required for patients with suspected or confirmed mycoses. Likewise, the use of antifungals for prophylaxis, empirical treatment, and long-term therapy may result in sub-therapeutic or toxic drug levels, which can lead to complications in patients. Therefore, the inadequate management of these medications and the growing emergence of antifungal-resistant *Aspergillus* strains are factors that have made this fungal infection a global concern. For this reason, a combined diagnostic approach is necessary [3,9].

The introduction of non-culture-based diagnostic tests, such as the detection of biomarkers, offers a non-invasive, accurate, and rapid alternative that may be useful for the early diagnosis of the disease, helping to confirm or rule out infection in patients receiving empirical treatment [9,10,11]. These biomarkers are also helpful in determining the duration of antifungal therapy and in identifying therapeutic and prophylactic failures [10,11]. The objective of this review is to describe several biomarkers and their usefulness in the diagnosis of aspergillosis.

## 2. *Aspergillus* spp.

Infection by *Aspergillus* spp. occurs through the inhalation of fungal conidia that circulate in indoor and outdoor environments [4]. These conidia enter the lungs and lodge in the lower airways due to their small size, measuring 2–3 μm in diameter. This small size allows the conidia to evade barrier mechanisms such as mucociliary clearance [12]. In immunocompetent individuals, conidia are cleared by the pulmonary macrophages, recruited neutrophils, and innate mechanisms such as the complement system [3,12]. Furthermore, these hosts develop a robust adaptive immune response characterized by the generation of memory T cells and B cells. In contrast, immunosuppressed individuals, whether due to an underlying condition or immunosuppressive therapy, are unable to contain the infection. Among individuals with therapy-induced immunosuppression, prolonged and sometimes profound neutropenia occurs and is recognized as the main risk factor for developing aspergillosis [3,12]. In addition to exposure and inhalation of fungal conidia, the development of aspergillosis depends on several factors: (*i*) the virulence of the *Aspergillus* strain, (*ii*) the individual’s immune status, and (*iii*) the host’s pulmonary structure and function. These factors determine the pathogenic mechanisms and the type of clinical manifestation [4,5].

The genus *Aspergillus* possesses various virulence-associated factors that are involved in several biological processes of the fungus, including water and nutrient balance, siderophore secretion, resistance to hypoxia, and cell wall resilience. The components of the fungal cell wall function not only as a protective barrier but also as a major source of ligands that activate and modulate the host’s immune response. For example, rodlets serve to mask highly immunogenic molecules to prevent recognition by the immune system. Additionally, this genus has the ability to form biofilms, in which galactosaminogalactan plays a prominent role, also functioning as a platelet activator. Likewise, the production of secondary metabolites such as gliotoxin and melanin enables the fungus to survive in extreme environments [6,8]. *A. fumigatus* is the species most frequently associated with human disease, serving as the etiologic agent in approximately 90% of aspergillosis cases, followed by *A. flavus*, *A. terreus*, and *A. niger* in decreasing order of frequency [3,8].

### 2.1. Clinical Presentations of Pulmonary Aspergillosis

Pulmonary aspergillosis encompasses a group of invasive and non-invasive diseases that affect the lungs. These conditions can be easily overlooked because they are often mistaken for other pulmonary diseases of infectious or non-infectious origin; therefore, aspergillosis should be considered as a differential diagnosis when clinically suspected [5]. Below, we describe the clinical forms, affected populations, signs and symptoms, and diagnostic criteria for allergic bronchopulmonary aspergillosis (ABPA), chronic pulmonary aspergillosis (CPA), and invasive aspergillosis (IA) (Table 1).

### 2.2. Allergic Bronchopulmonary Aspergillosis (ABPA)

ABPA is a disease characterized by a type I hypersensitivity reaction induced by *Aspergillus* antigens. The population affected includes atopic individuals, most of whom also have cystic fibrosis or asthma [4,13]. However, cases have also been documented in patients with chronic obstructive pulmonary disease (COPD), a history of tuberculosis, and lung transplant recipients [5]. Recent estimates indicate that this disease occurs in approximately 4.8 million cases annually worldwide [2]. Prevalence is estimated at 1–3% among patients with asthma and 10–9% among adult and pediatric patients with cystic fibrosis, respectively [13]. In these patients, the fungus is able to evade innate immune mechanisms and subsequently induce a Th2-type CD4+ T-cell response, triggering production of Th2 cytokines, IgE, and activation of mast cells in tissues—features typical of a type I hypersensitivity or allergic reaction to *Aspergillus* antigens [5].

### 2.3. Chronic Pulmonary Aspergillosis (CPA)

CPA is considered a group of progressive, non-invasive fungal diseases. It can affect immunocompetent patients or those with mild immunosuppression, particularly individuals with pre-existing pulmonary conditions such as emphysema, COPD, or sequelae of tuberculosis [5]. This condition is associated with impaired mucociliary clearance and dysregulated inflammatory responses that contribute to disease severity [4]. Although the true prevalence is unknown, it is estimated that approximately three million cases occur worldwide each year [2]. It is also estimated that more than 350,000 annual cases of CPA associated with tuberculosis become complicated within 12 months after completion of anti-tuberculosis treatment [4]. This disease spectrum has a poor prognosis, as mortality during the first five years can reach up to 80% [14].

### 2.4. Invasive Pulmonary Aspergillosis (IPA)

IPA is the most severe clinical form of aspergillosis. It is associated with immunosuppression due to chemotherapy and corticosteroid administration that leads to neutropenia, as well as with HIV infection and inborn errors of immunity [3]. It also includes patients with acute viral pneumonias who develop influenza-associated pulmonary aspergillosis (IAPA) and COVID-19-associated pulmonary aspergillosis (CAPA) [15,16,17]. Historically, hematopoietic stem cell transplant (HSCT) recipients were the primary population affected; however, this clinical presentation has increasingly been reported in non-neutropenic patients [5]. IPA has also been described in other patient groups, such as solid organ transplant recipients and individuals with diabetes mellitus or liver disease, where underlying conditions, immunosuppressive therapies, and comorbidities contribute to risk [4].

Surveillance of this disease is highly relevant because it is among the costliest conditions for healthcare systems due to prolonged hospitalization, treatment expenses, and the high costs associated with managing complications [3]. It is estimated that approximately 250,000 cases of IPA occur annually worldwide. IPA is an acute disease with rapid progression over days to weeks [2], and is associated with high mortality rates ranging from 40% to 50% in patients with acute leukemia and HSCT recipients [4].

Invasive pulmonary aspergillosis (IPA) involves hyphal invasion of the bronchial wall and blood vessels [18]. Clinical manifestations are nonspecific and may mimic bronchopneumonia, with possible chest pain and hemoptysis. Angioinvasion can lead to thrombosis and hemorrhagic infarction, and hematogenous dissemination occurs in about 25% of cases [5,18]. Viral pneumonia further predisposes patients through epithelial damage and immune dysfunction [16,17].

The EORTC/MSG (European Organization for Research and Treatment of Cancer/Mycoses Study Group) has established a series of criteria and recommendations for clinical research, diagnosis, and epidemiology to define IPA cases. The criteria for proven cases apply to both immunocompetent and immunocompromised individuals, whereas the criteria for probable and possible cases apply only to immunocompromised individuals. Possible cases meet clinical criteria and have a host factor but lack microbiological evidence, distinguishing them from probable cases (Table 2) [15]. These recommendations are also incorporated into the Colombian consensus on the diagnosis and management of *Aspergillus* disease and invasive aspergillosis in adult and pediatric patients [15,19].

**Table 2:** Case definition of invasive aspergillosis (IA) [1,10,15,19,20,21].

**Proven IA**	Histopathological, Cytopathological, or Microscopic Examination of a Specimen Obtained by Needle Aspiration or Biopsy Showing Hyphae Evidence of Associated Tissue Damage.
**Probable/Possible IA**	At least one of the following host factors: Recent history of neutropenia lasting > 10 days temporally related to the onset of invasive fungal disease (absolute neutrophil count ≤ 500 cells/mm^3^). Hematologic malignancy. Receipt of allogeneic stem cell transplant. Receipt for solid organ transplant. Prolonged corticosteroid use (except in ABPA patients) at a therapeutic dose ≥ 0.3 mg/kg for ≥3 weeks within the last 60 days. Treatment with other T-cell immunosuppressants within the last 90 days. Treatment with B-cell immunosuppressants. Severe inherited immunodeficiency. Acute grade III–IV graft-versus-host disease involving gut, lungs, or liver, refractory to first-line steroid therapy. *At least one of the following clinical or radiological features of pulmonary infection:* Dense, well-defined lesions with or without halo sign. Air crescent sign. Cavity. Wedge-shaped, segmental, or lobar consolidation. *At least one of the following mycological criteria:* Positive cytology, microscopy, and/or culture for *Aspergillus* from lower respiratory tract samples (sputum, BAL, bronchial brush, or aspirate). *Aspergillus* galactomannan antigen index: - Single serum or plasma sample ≥ 1.0. - BAL fluid ≥ 1.0. - Single serum or plasma ≥ 0.7 and BAL fluid ≥ 0.8. *Aspergillus* PCR, one of the following conditions: - ≥2 consecutive positive PCR tests in plasma, serum, or whole blood. - ≥2 duplicate positive PCR tests in BAL. - ≥1 positive PCR in plasma, serum, or whole blood and ≥1 positive PCR in BAL.

For IAPA and CAPA, the above-mentioned criteria do not fully apply because these conditions may present with atypical clinical and radiological features that are not typical of invasive fungal disease [17], Therefore, alternative consensus definitions for CAPA, intended for research use, have been proposed by ECMM (European Confederation of Medical Mycology) and ISHAM (The International Society for Human & Animal Mycology) [16].

## 3. Biomarkers

A biomarker is defined as a biological product or component of a microorganism that enables its identification and the early diagnosis of disease [11]. Some of the biomarkers associated with the diagnosis of aspergillosis are described below. Biomarkers are classified into three categories: cell wall components, specific genetic sequences, and metabolism-derived products.

### 3.1. Tests for the Detection of Anti-Aspergillus Antibodies

In CPA and ABPA, the affected population consists of immunocompetent patients or those with mild immunosuppression who are capable of producing antibodies effectively. In these clinical presentations of aspergillosis, antibody-based biomarkers play an important diagnostic role. Available anti-*Aspergillus* antibody tests include the precipitin test and the detection of IgG, IgA, IgM, and IgE levels. The European Society of Clinical Microbiology and Infectious Diseases (ESCMID) and the European Respiratory Society (ERS) guidelines recommend IgG detection and the precipitin test for the diagnosis of CPA, whereas IgA and IgM antibodies against *Aspergillus* are not recommended in these guidelines nor mentioned in the IDSA (Infectious Diseases Society of America) guidelines due to insufficient relevant data [1,20,22]. A meta-analysis assessing the diagnostic accuracy of these antibodies for CPA found that IgG had superior diagnostic performance compared with the others; IgA showed moderate performance, while the precipitin test and IgM alone and IgG + IgM were inferior [14,23,24]. In the case of ABPA, the disease is characterized by elevated levels of *Aspergillus*-specific IgE and IgG due to type I and type III hypersensitivity reactions [25], and these markers are used in its diagnosis [4] (Table 1). However, antibody levels, production kinetics, and antibody isotypes have not been correlated with disease severity. Therefore, it is necessary to develop standardized methods for quantifying anti-*Aspergillus* antibody titers and correlating their values with disease activity, severity, and response to therapy, particularly in CPA [4].

### 3.2. Galactomannan (GM)

It has been the most extensively studied biomarker, particularly in patients with hematological malignancies, and its determination has become one of the major diagnostic innovations in medical mycology in recent decades [26]. This component has been the antigen of choice for the diagnosis of IA since its discovery in 1970, as it is released during fungal growth (angioinvasion) [4]. Consequently, the level of this antigen in body fluids such as bronchoalveolar lavage (BAL) and serum is associated with fungal burden and active infection [27]. Likewise, the side chain composed of galactofuranose confers immunogenicity and induces the production of monoclonal antibodies with high affinity and avidity [4].

Galactomannan is composed of an α-(1,2)(1,6)-mannopyranose backbone with short β-(1,5)-oligogalactofuranose side chains linked by β-(1,3) and β-(1,6) bonds [28]. This polysaccharide is an important component of the cell wall of filamentous fungi and, although it is present in lower proportions compared with other components such as α-glucans, it forms part of the central core of the cell wall. This core is composed of branched β-(1,3)(1,6)-glucan that is linked to chitin and galactomannan. In hyphae, the alkali-insoluble inner cell wall fraction contains 5% GM, whereas the alkali-soluble outer cell wall fraction contains 1.4% GM [29]. Although a homolog has recently been identified in the conidial cell wall, it is composed of longer galactofuranose side chains with 6-O substitutions [30]. GM is also considered to be preferentially recognized by mannose receptors such as DC-SIGN, ICAM-3, the mannose-binding lectin, and Dectin-2 [28].

The detection of this molecule in serum and BAL has been included in the guidelines of the ESCMID and the ECMM (European Confederation of Medical Mycology) and has been accepted by the ERS and the EORTC/MSG as complementary diagnostic assays for the diagnosis of aspergillosis [15,20]. Because it is a circulating antigen, serial determinations are recommended. In addition, it can be used as a monitoring biomarker in specific populations, such as patients with hematological malignancies and HSCT recipients. GM determination is also useful for evaluating disease progression and therapeutic response [1].

The methodologies available for the detection of this molecule include enzyme-linked immunosorbent assay (ELISA), lateral flow assay (LFA), and chemiluminescence immunoassay, all of which can be performed using serum and bronchoalveolar lavage samples. ELISA has limitations such as assay turnaround time (approximately 3 h), the need for trained personnel, and laboratory equipment [31]. Currently, LFA is marketed in several countries worldwide and consists of a simple, single-test device that enables faster detection without the need for complex equipment. Some LFAs incorporate portable optical devices that measure the intensity of the test bands, allowing quantification of the analyte of interest [32]. Chemiluminescence immunoassays are also available in a single-test format and provide results in approximately 1 h; however, they require specialized instruments for execution [33] (Table 3). Across the methodologies mentioned above, false-positive results may occur, mainly associated with patient-related factors such as bacteremia, multiple myeloma, dialysis, and a high-fiber diet, or due to cross-reactivity with GM epitopes from other fungal genera such as *H. capsulatum*, *T. marneffei*, and *Fusarium* spp. [31,34,35] (Table 4).

Several studies have evaluated the diagnostic performance of GM detection in IA, reporting a sensitivity ranging from 48% to 90% and specificities from 80% to 100%. This analytical performance depends on the type of specimen, the patient population, and underlying conditions. In general, GM detection has been shown to perform better in BAL samples in cases of infection confined to the lungs, whereas in disseminated infection, serum antigen detection is more useful [4,36,37,38,39]. A meta-analysis in hospitalized immunocompromised patients, including hematologic patients and those with neutropenia, reported a sensitivity of 78% and a specificity of 85% when using a cutoff ≥ 0.5 on serum samples. As the cutoff value increased, sensitivity decreased while specificity increased [39]. These findings are similar to those reported in another study of immunocompromised patients with hematological malignancies using a cutoff ≥ 0.5, which found a sensitivity of 80% and a specificity of 81% [40]. Additionally, these differences may also be associated with the type of sample. In a study of lung transplant recipients, higher sensitivity was observed in bronchoalveolar lavage (BAL) samples compared to serum samples (cutoff ≥ 0.5; serum sensitivity: 84%, specificity: 80%; cutoff ≥ 1.0 BAL sensitivity 92.6% and specificity 88.6%) [41].

**Table 3:** Commercial assays available for the detection of biomarkers in aspergillosis.

Target	Assay Reference	Assay Type	Specimen	Manufacture
Antibodies	*Aspergillus fumigatus* IgA ELISA Kit	ELISA	Serum	QED Bioscience Inc.
	*Aspergillus* IgG Antibody Detection K-Set	Lateral flow assay	Serum	Era Biology
	*Aspergillus* IgM Antibody Detection K-Set	Lateral flow assay	Serum	Era Biology
	*Aspergillus fumigatus* IgG ELISA	ELISA	Serum	Demeditec
	*Aspergillus fumigatus* IgG ELISA Kit	ELISA	Serum	Aviva Systems Biology
	*Aspergillus fumigatus* IgG ELISA Kit	ELISA	Serum	QED Bioscience Inc.
	*Aspergillus fumigatus* IgE ELISA	ELISA	Serum	Demeditec
	*Aspergillus fumigatus* IgM ELISA Kit	ELISA	Serum	Aviva Systems Biology
	*Aspergillus fumigatus* IgM ELISA Kit	ELISA	Serum	QED Bioscience Inc.
	Dynamiker *Aspergillus fumigatus* IgG	ELISA	Serum	Dynamiker
	QuicIgG *Aspergillus* IgG Ab Lateral Flow Assay	Lateral flow assay	Serum	Dynamiker
Galactomannan	*Aspergillus* Galactomannan Ag VIRCLIA	Sandwich chemiluminescent immunoassay	Serum and BAL	Vircell S.L.
	*Aspergillus* Galactomannan Detection K-Set	Lateral flow assay	Serum and BAL	Era Biology
	Dynamiker *Aspergillus* Galactomannan Assay	ELISA	Serum and BAL	Dynamiker
	FungiXpert^®^ *Aspergillus* Galactomannan	ELISA	Serum and BAL	Era Biology
	*Aspergillus* Galactomannan (CLIA)	Chemiluminescence immunoassay	Serum and BAL	Era Biology
	Platelia *Aspergillus* Ag	ELISA	Serum and BAL	Bio-Rad
	Sōna *Aspergillus* GM Lateral Flow Assay	Lateral flow assay	Serum and BAL	IMMY
	QuickGM *Aspergillus* Galactomannan Ag	Lateral flow assay	Serum and BAL	Dynamiker
Mannoprotein	*Aspergillus* Antigen ELISA	ELISA	Serum and BAL	EUROIMMUN
1,3-β-D-glucan	Beta-Glucan Test	Enzymatic reaction	Serum	FUJIFILM
	Dynamiker Fungus (1-3)-β-D-Glucan	ELISA	Serum	Dynamiker
	Goldstream^®^ Fungus (1-3)-β-D-glucan Test (available in China and Europe)	Enzymatic reaction	Serum	Era Biology
	Fungitell Assay	Enzymatic reaction (single test)	Serum	Fungitell
	Fungitell STAT Assay	Enzymatic reaction	Serum	Fungitell
DNA detection	AsperGenius	Real-time PCR (species ID and azole resistance)	BAL	PathoNostics
	GeneProof *Aspergillus* PCR Kit	Real-time PCR (ITS2/28S rDNA)	Whole blood, plasma, serum, BAL, sputum, CSF	GeneProof
	Goldstream^®^ *Aspergillus* Molecular Test (China and Europe)	Real-time PCR	Whole blood and BAL	Era Biology
	MycAssay *Aspergillus*	Real-time PCR	Lower respiratory tract samples and serum	Myconostica UK
	MycoGENIE^®^ *Aspergillus fumigatus* and resistant TR34/L98H	Real-time PCR	Serum, biopsy, lower respiratory tract samples	Ademtech
	MycoGENIE *Aspergillus* species—Mucorales species	Real-time PCR	Serum, biopsy, lower respiratory tract samples	Ademtech
	VIASURE *Aspergillus* differentiation Real Time PCR Detection Kit	Real-time PCR (*A. fumigatus*, *A. flavus*, *A. terreus*)	BAL, bronchial aspirate, sputum	Certest

**Table 4:** Advantages and disadvantages of the use of biomarkers in clinical diagnosis.

Advantages	Disadvantages
Faster diagnosis leads to more accurate clinical and therapeutic management.	Low biomarker concentration could degenerate false negative results.
Indirect evidence of the presence of the microorganism in the specimen.	Limited knowledge about the kinetics of biomarker release.
Assay adaptable to different specimen types.	Lack of awareness among clinicians regarding new technologies and diagnostic alternatives.
Biomarkers are used as indicators for therapeutic monitoring and prognosis.	Limited global availability of commercial kits.
Combination of biomarkers allows more accurate diagnosis.	Risk of cross-reactivity, especially with genetically closely related microorganisms.
References: [21,36,42,43,44,45,46]	

In COVID-19-associated pulmonary aspergillosis (CAPA), positive results have been obtained in approximately 20% of serum samples, but in more than 80% of respiratory samples [47,48]. The performance of antigen detection in urine, cerebrospinal fluid (CSF), and other body fluids remains under investigation. It is important to note that, at the time of writing this review, commercially available reagent kits specify only serum and BAL as validated sample types [10,39]. Low sensitivity rates have been associated with pediatric patients, non-neutropenic patients, solid organ transplant recipients, patients with low fungal burden, and those receiving antifungal prophylaxis [4]. In studies conducted in ICU patients with COVID-19 using serum samples and a cutoff value of 0.5, sensitivity ranged from 20% to 40% and specificity from 82% to 93% [49,50]. In contrast, a systematic review in patients with COVID-19-associated pulmonary aspergillosis (CAPA) using bronchoalveolar lavage (BAL) samples and a cutoff value of 1.0 reported a sensitivity of 81.3% and a specificity of 79.5% [51]. In the latter case, antifungal agents inhibit fungal growth and reduce fungal burden, thereby limiting infection and decreasing the amount of GM released into the circulation [15].

GM detection has also been evaluated in patients with CPA. Serum ELISA has shown low sensitivity and specificity, even when the index cutoff value is reduced from 1.5 to 0.5 (the recommended cutoff for the Platelia *Aspergillus* Ag assay, Bio-Rad, Hercules, CA, USA). Studies have reported a BAL sensitivity and specificity of 77%, slightly higher than those observed in serum [35]; In another study that included patients with APC, aspergilloma, AI, and CAPA, a sensitivity of 89% and a specificity of 75% were obtained [52]. In another study that included patients with CPA, the optimal cutoff value is 0.55, a sensitivity of 38% and a specificity of 87% [53]. Mean GM values may be higher in patients with CPA than in those with ABPA, which may be related to pulmonary damage and pre-existing conditions affecting lung structure and function that facilitate fungal colonization, whereas ABPA is characterized by a generalized inflammatory response in the lungs due to the presence of the fungus [27].

### 3.3. Mannoprotein

As described above, the fungal cell is rich in mannose, and many of its structural proteins are mannosylated. These form a glycoconjugate known as mannoproteins, which, like GM, are released when the fungus is actively growing. The role of certain mannoproteins as antigens for immunodiagnosis has been investigated, in addition to their role in inducing T cell-mediated immunity [54]. The synthesis of these compounds begins with the extension of the mannose chain by the addition of at least 10 residues linked by α-1,6 bonds, to which mannose and phosphomannose residues are subsequently added through α-1,2 or α-1,3 linkages. This molecule is transported to the extracellular space, where it covalently binds to β-glucan to form the outermost layer of the fungal cell wall [55].

At the commercial level, an LFA-based test for the detection of this antigen is available, making it a simple-to-use device that provides rapid results (Table 3). The performance of this assay in BAL samples has shown a sensitivity close to 70% and a specificity of 100% in patients at risk for IA [56]. A meta-analysis concluded that the diagnostic performance of this test in BAL samples was superior to that in serum samples, with a sensitivity of 86% and a specificity of 93%. As with GM, cross-reactivity has been reported with *Penicillium* spp., *Histoplasma capsulatum*, and *Fusarium oxysporum* [32].

### 3.4. Beta-1,3-D-Glucan (BDG)

β-Glucan is a linear polysaccharide composed of glucose monomers linked by β-(1,3) bonds. This compound is synthesized at the plasma membrane and serves as an essential component in maintaining the structural integrity of the cell wall, as well as an anchoring point for other polymers [57]. In conidia, it is found in the soluble fraction of the outer cell wall at approximately 5%, where it forms a network of macromolecules that, because they are not covalently bound, are more flexible. In contrast, in the inner portion of the cell wall, it is present in higher proportions (38%). In hyphae, β-glucan is in the insoluble fraction of the inner cell wall, accounting for approximately 30% [58].

After synthesis, several modifications occur that remodel polysaccharides through specific hydrolases and a glucosyltransferase. These polysaccharides become cross-linked, forming a network through their association with chitin, galactomannan, and proteins, which confers rigidity and strength to the inner layer of the cell wall [29,58]. BDG is a PAMP (pathogen-associated molecular pattern) that is recognized by Dectin-1 during infection. This receptor is expressed on different cells of the immune system [59]; however, in resting conidia, BDG is masked by rodlet layers. When conidia begin to germinate, their structure changes and enlarges. The rodlets then detach from BDG, exposing it; nevertheless, it is subsequently covered again by a layer of galactosaminogalactan. This stage is thought to be important for immune recognition, during which leukocyte recruitment to the lungs occurs in response to fungal infection [60,61].

For the in vitro detection of BDG, the ability of this antigen to activate the coagulation cascade through interaction with factor G of the horseshoe crab (*Limulus* spp.) is exploited. Subsequently, this serine protease activates a clotting enzyme, which in turn activates a substrate that can be detected in the assay. Available assays differ in the artificial substrate used [62]. It is important to note that this antigen is not specific to a single genus or species, as it is produced by the genus *Aspergillus* spp., *Candida* spp., *Pneumocystis* spp., *Histoplasma* spp., and *Coccidioides* spp., among others. *Cryptococcus* spp., *Blastomyces* spp., and the mucorales do not express glucans detectable by this method [11]. Nevertheless, although it is a panfungal detection method, in the case of aspergillosis, a sensitivity greater than 60% has been documented [63].

Additionally, sensitivity and specificity rates have been reported to range from 67% to 100% and from 50% to 100%, respectively [4,39,64]; however, the diagnostic value depends on disease prevalence. In a meta-analysis of patients with suspected invasive aspergillosis, using a cutoff of 80 pg/mL, a sensitivity of 72% and a specificity of 82% were reported [65]. In some cases, its use is not recommended in patients receiving antifungal prophylaxis due to a low positive predictive value [39]. Several commercial kits are available for BDG determination, with variations in performance because they are based on different methods and use reagents obtained from different genera of horseshoe crabs; consequently, their cutoff values also vary (Table 4). Some manufacturers have focused on improving assay specificity by using alternative reagents that react exclusively with BDG polysaccharides and not with other polysaccharides containing different glycosidic linkages in their structure [62]. Its diagnostic value lies in the fact that, although it is not specific for *Aspergillus*, its use has been recommended as a complementary test for the diagnosis of IA in high-risk patients (e.g., those with hematological malignancies and HSCT recipients) [1]. It has also been documented that BDG tests may become positive earlier than GM tests [37] and can precede clinical manifestations by approximately ten days in patients with invasive fungal infections [11]. Furthermore, the negative predictive value of BDG has been shown to reduce inappropriate antifungal use by up to 90% in immunocompromised patients and those in intensive care units (ICUs) [66].

Finally, some of the platforms used for BDG determination have limitations, including the need for trained personnel, lengthy assay times (approximately 3 h), and specialized laboratory equipment, which in turn requires strict environmental control to avoid exogenous contamination. This test requires a small sample volume (10–70 μL of serum), and newer platforms have been developed that can reduce processing time by up to 2 h. Causes of false-positive results include hemodialysis with cellulose membranes, administration of blood products through filters, bacteremia caused by *Streptococcus* spp. or Gram-negative bacilli, tissue contact with cotton gauze, and the administration of antibiotics such as amoxicillin–clavulanate and piperacillin–tazobactam. False-negative results have been associated with antifungal prophylaxis and with sera containing high concentrations of bilirubin and triglycerides [11]. Currently, the importance of incorporating this test in healthcare centers that manage immunocompromised patients is emphasized, as it has been included in the ESCMID and ECMM guidelines and accepted by the ERS as a complementary diagnostic technique. In addition, it is part of the Colombian consensus on the diagnosis and follow-up of *Aspergillus* disease and invasive aspergillosis in adult and pediatric patients [11,15,19,20].

### 3.5. DNA Sequences

The genomes of *Aspergillus* species consist of eight chromosomes and range in size from approximately 28–40 Mb [67]. *A. fumigatus* is the species most frequently associated with pulmonary infection in humans; it has a 29 Mb genome comprising 9926 genes [68]. Common molecular diagnostic targets include the ribosomal 18S and 28S genes and the internal transcribed spacer (ITS) region. Their high conservation enables the use of universal primers to detect unknown species, while their high copy number—10 to 100 times greater than single-copy genes—enhances analytical sensitivity [69,70]. Although these regions are frequently used, other molecular targets are crucial for *Aspergillus* taxonomy, such as β-tubulin (benA, tub-2) and calmodulin (caM). When combined with ITS regions, these targets enable accurate identification of species within this genus [71].

Polymerase chain reaction (PCR) has been widely used as a diagnostic methodology in aspergillosis, particularly in IA [69], and has been included in the EORTC/MSG guidelines as a microbiological criterion for the diagnosis of probable IA [15]. *Aspergillus* DNA has been detected in various types of clinical samples, including blood, BAL, and sputum [27]. Test performance depends on the type of specimen, the population studied, and the PCR protocol, including the type of PCR assay and the molecular target used. For qPCR, amplification of the ITS region from blood samples has been reported to yield sensitivities ranging from 44% to 80% and specificities from 57% to 100% [72,73,74]. A multicenter study in hematologic patients with proven/probable invasive aspergillosis reported a sensitivity of 68.4% and a specificity of 76.2%; sensitivity of 94.7% and specificity of 83.3% in serum and plasma samples, respectively [74]. and in BAL sensitivity of 64% and specificity of 99% in patients with suspected aspergillosis [75].

Other studies have shown that qPCR targeting the 28S rRNA gene in blood samples achieved sensitivity between 69% and 95% and specificities between 36% and 73% [76,77]. In contrast, qPCR performed on BAL samples targeting the 18S rRNA gene has reported sensitivity and specificity values of 70% and 100%, respectively [78].

The development of commercial PCR kits for *Aspergillus* has contributed to assay standardization. These platforms have been validated in BAL and other respiratory samples, across populations with different underlying conditions, and demonstrate sensitivities of 80–94% and specificities of 97–99% (Table 3). In addition, PCR assays capable of detecting azole resistance markers in the *cyp51A* gene of *A. fumigatus* have been developed. However, organizations such as the Infectious Diseases Society of America recommend that *Aspergillus* PCR be used on a case-by-case basis and as a complement to other clinical and diagnostic data [9]. To provide validated and standardized PCR protocols for *Aspergillus* in clinical practice, the European *Aspergillus* PCR Initiative (EAPCRI) was launched in 2006 [79]. This initiative has validated PCR assays using serum, plasma, and whole blood samples and is currently working on the validation of assays in BAL. Information on other sample types, such as urine or CSF, remains limited [80].

Overall, comparative studies have shown that PCR is more sensitive than culture in blood and respiratory fluids. PCR in blood or serum alone cannot confirm a diagnosis of IA, and although PCR sensitivity is higher in BAL than in blood or serum, its specificity is lower due to respiratory tract colonization by *Aspergillus*, particularly in high-risk populations such as lung transplant recipients, in whom PCR cannot reliably discriminate between infection and colonization [1]. In general, the use of this test is not recommended in patients receiving prophylactic or empirical antifungal therapy, as such treatment may reduce the fungal burden below the limits of detection [80].

Peptide nucleic acid fluorescence in situ hybridization (PNA-FISH) is a rapid molecular method that enables direct identification of *Aspergillus* spp. through hybridization of fluorescently labeled PNA probes to species-specific ribosomal RNA targets. Assays have been developed to detect the genus *Aspergillus* and clinically relevant species, particularly members of the *Aspergillus fumigatus* complex, as well as *Aspergillus flavus* and *Aspergillus terreus*. The method can be applied directly to respiratory samples, tissue sections, or culture isolates, providing results within a few hours. Although PNA-FISH does not provide antifungal susceptibility or resistance data and requires fluorescence microscopy infrastructure, it represents a valuable adjunct diagnostic tool for the rapid species-level identification of *Aspergillus* in patients at risk for invasive aspergillosis [81,82].

Beyond species identification, molecular assays have increasingly been developed to detect antifungal resistance, particularly azole resistance in *A. fumigatus*, which is most commonly associated with mutations in the CYP51A gene and its promoter region (e.g., TR34/L98H and TR46/Y121F/T289A). The ability to identify these mutations directly from clinical specimens, especially BAL, has important clinical implications, as azole-resistant infections are associated with higher rates of treatment failure and mortality and may necessitate early modification of antifungal therapy to alternative agents such as liposomal amphotericin B. Commercial real-time PCR platforms that simultaneously detect *A. fumigatus* DNA and common CYP51A resistance mutations represent a significant advance toward rapid, culture-independent resistance detection. However, several challenges remain. Not all resistance mechanisms are mediated by CYP51A mutations, and rare or emerging mutations may not be covered by current assays, potentially leading to false-negative resistance results. In addition, the sensitivity of resistance detection depends on fungal burden, sample quality, and the presence of mixed susceptible and resistant populations. The limited availability of standardized resistance panels, variability in assay targets, and the need for continuous updating of molecular platforms to reflect evolving resistance patterns further complicate implementation. Regulatory approval and demonstration of clinical utility in large prospective studies are also required. Therefore, while PCR-based resistance detection is a promising tool for guiding antifungal stewardship, it should currently be integrated with culture, susceptibility testing, and clinical assessment to optimize patient management [81,82].

## 4. Metabolic Derivatives

### 4.1. Triacetylfusarinine C (TAFC)

During infection, *Aspergillus* produces low-molecular-weight iron chelators, known as siderophores, which play a fundamental role in the virulence of the microorganism [83,84]. Triacetylfusarinine C (TAFC) is the main siderophore of *A. fumigatus* and *A. nidulans*; therefore, it is not a biomarker for all *Aspergillus* species. In addition, it is noteworthy that this biomarker is not produced by mucoralean fungi or by bacteria [83]. TAFC is a potential early diagnostic biomarker for IA, as it is secreted only during active fungal growth and is not present in conidia [83,85]. It may also be important for assessing treatment prognosis [83].

TAFC has been detected in human body fluids, specifically in BAL, serum, and urine. Its detection relies on methodologies such as high-performance liquid chromatography (HPLC), capillary electrophoresis–electrospray ionization–mass spectrometry (CE–ESI–MS), and liquid chromatography–mass spectrometry (LC–MS/MS) [83,84,85]. In an initial study, a sensitivity of 81% and a specificity of 90% were reported in urine samples from patients with hematological malignancies using CE–ESI–MS, with better performance than that observed in serum and BAL samples. This has been suggested to be explained by the accumulation of the siderophore in the bladder, as observed in in vivo models [83].

Because the diagnostic techniques used in previous studies are costly and require specialized equipment, appropriate facilities, and highly trained personnel, efforts have been made to generate monoclonal antibodies against TAFC. The aim is to enable their use in simpler platforms that would allow the development of laboratory tests at the point of care (POC) [84].

This siderophore also has the ability to chelate radioactive metals that are recognized by siderophore transporters, which could be useful for in vivo imaging of *Aspergillus* infection [83,84]. Evaluations in animal models have demonstrated its potential as a non-invasive diagnostic tool [83].

### 4.2. Volatile Organic Compounds (VOCs)

Volatile organic compounds (VOCs) induced by *Aspergillus* are characterized by a carbon-rich backbone, low molecular weight, and represent byproducts of metabolic processes. They can be obtained from various samples, including blood, saliva, breath, and urine, among others. During disease, these VOCs may be produced specifically as a result of metabolic alterations and can be detected using advanced techniques such as gas chromatography coupled to mass spectrometry (GC–MS) [86]. VOCs have been used as screening tools in other diseases, such as cancer, chronic obstructive pulmonary disease (COPD), and cystic fibrosis [87]. They have also been applied in the construction field to detect sick building syndrome, where these compounds provide an indication of excessive growth of molds such as *Mucor* spp., *Aspergillus* spp., and *Fusarium* spp. [88].

Among the VOCs that can be identified is 2-pentylfuran (2PF), which has been associated with aspergillosis. This molecule is small, volatile, poorly soluble in water, and is produced exclusively by filamentous fungi; therefore, it lacks diagnostic specificity [42,86]. Because these compounds are exhaled, they have the potential to be readily detected in the breath of patients with aspergillosis, making this an attractive approach due to the simplicity of sample collection. Currently, some studies using this technique report sensitivity and specificities of approximately 80% to 100%; however, additional studies are required to better understand the performance of these assays [42,43].

Although TAFC and VOCs show considerable promise as innovative biomarkers, they should currently be regarded as experimental or emerging tools rather than established diagnostic modalities. Several important challenges must be addressed before their routine clinical implementation. First, the detection of TAFC and most VOCs depends on advanced analytical platforms such as LC–MS/MS, CE–ESI–MS, or GC–MS, which are costly, technically demanding, and limited to specialized laboratories. This restricts accessibility, particularly in low- and middle-income settings where invasive aspergillosis is highly prevalent. Second, there is a lack of standardized protocols for sample collection, processing, storage, and analysis, leading to variability across studies and limiting reproducibility and inter-laboratory comparability. Third, clinically validated cut-off values and large prospective multicenter studies are still lacking, which hampers the establishment of clear diagnostic algorithms and performance benchmarks. In addition, regulatory approval pathways for metabolite-based assays require rigorous analytical and clinical validation, as well as demonstration of clinical utility and cost-effectiveness. Future efforts should therefore focus on assay harmonization, simplification of detection platforms (e.g., antibody-based or point-of-care formats), robust clinical validation in well-defined patient populations, and integration with existing fungal diagnostic strategies to facilitate their translation into routine practice [42,43,83,84,85,86,87,88].

### 4.3. Complementarity of Biomarkers

Several studies have reported that combining biomarkers considerably improves diagnostic sensitivity and/or specificity, particularly in the case of IA. The potential of these tests when used individually has a limited role [9]. The combined use of GM and BDG during the early phases of infection has been highlighted; one study reported an increase in sensitivity from 60% to 83% when this combined detection was applied in hematological patients, in addition to being a non-invasive tool [11,26]. The combination of GM level detection and *Aspergillus* sequence copy number has been described as the best biomarker combination strategy for the diagnosis of IA, as it increases sensitivity, especially in patients who develop IAPA or CAPA [4]. Furthermore, this strategy reduces antifungal use and has been associated with a lower incidence of IA and earlier diagnosis [1]. Other combinations have also been evaluated, such as the detection of GM and TAFC in BAL, which increases sensitivity from 73% to 87% [83].

**Figure 1:**
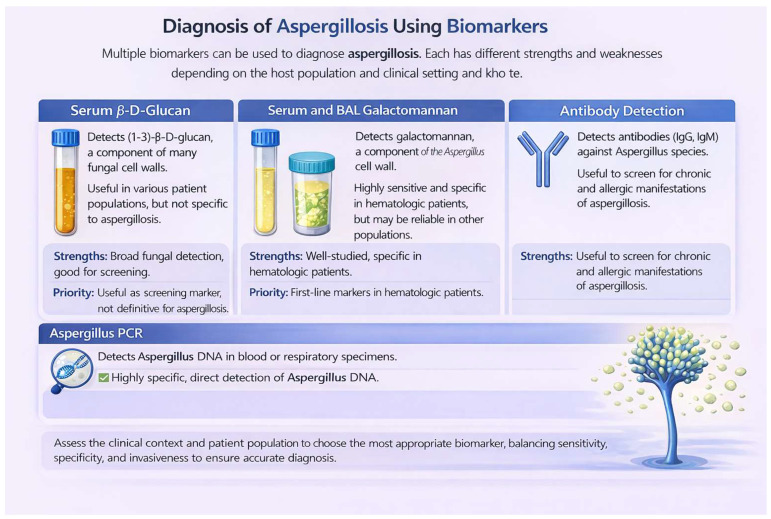
Biomarkers for the Diagnosis of Aspergillosis: Strengths, Limitations, and Clinical Prioritization.

**Figure 2:**
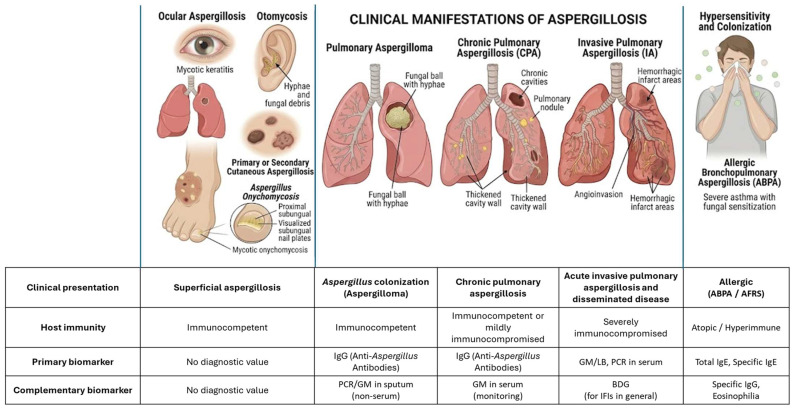
Recommended Diagnostic Approaches Across the Clinical Spectrum of Aspergillosis.

As described throughout this review article, biomarkers represent one of the most important diagnostic alternatives developed in recent years for the diagnosis of fungal diseases. The usefulness of their detection is determined by the patient’s underlying condition, the degree of immunocompromise, and the clinical form of aspergillosis, aspects that are summarized in Table 4. In summary, no single biomarker performs uniformly across all patient populations. Diagnostic strategies should be individualized based on immune status, suspected disease severity, and local epidemiology. A combined or sequential testing approach may optimize diagnostic accuracy while improving clinical outcomes (Figure 1 and Figure 2).

## 5. Conclusions

The detection of biomarkers using non-culture-based diagnostics represents one of the most important innovations of recent decades in the field of medical mycology. Biomarkers have opened new avenues for the management of fungal diseases by providing non-invasive, rapid, and accurate diagnostic support [11]. The identification of circulating molecules with potential diagnostic value as disease biomarkers and their subsequent implementation still present some challenges that remain to be resolved; however, recent advances in the understanding of the biology, genomics, proteomics, and transcriptomics of *Aspergillus* species have enabled the identification of additional potential biomarkers that will support the design of new diagnostic methods [75,76,77,78,79,80,81].

## Figures and Tables

**Table 1:** Clinical form, affected population, signs and symptoms, and diagnostic criteria for allergic bronchopulmonary aspergillosis (ABPA), chronic pulmonary aspergillosis (CPA), and invasive aspergillosis (IA).

Clinical Form	People at Risk	Signs and Symptoms	Diagnostic Criteria
Allergic bronchopulmonary aspergillosis (ABPA)	Cystic fibrosis or asthma.	Recurrent wheezing, expectoration of mucus plugs, worsening of asthma, recurrent pulmonary infiltrates.	Clinical, radiological, and immunological features. (1) Positive skin test to *A. fumigatus* antigens and/or elevated serum IgE antibodies to *A. fumigatus*. (2) Total serum IgE levels > 1000 U/mL plus supportive criteria (2 of 3 required): Elevated serum IgG antibodies to *A. fumigatus* or positive *Aspergillus* precipitins. Radiological findings consistent with ABPA. Peripheral blood eosinophil count > 500 cells/µL in patients without prior corticosteroid treatment.
Chronic pulmonary aspergillosis (CPA)	Immunocompetent patients or those with mild immunosuppression and pre-existing lung disease.	Chronic inflammation (>3 months) with weight loss, chronic productive cough, fatigue, and hemoptysis; imaging shows nodules, cavities, or fungal balls.	For at least 3 months: Presence of one or more cavities with or without nodules or fungal ball. Positive culture or microscopy for *Aspergillus*, or presence of *Aspergillus* IgG antibodies (positive in >90% of CPA patients).
Invasive aspergillosis (IA)	Primarily hematopoietic stem cell transplant (HSCT) recipients; cases also reported in other immunosuppressed patients. IAPA: Patients with influenza pneumonia who develop aspergillosis. CAPA: Patients with SARS-CoV-2 pneumonia who develop aspergillosis.	See Table 2.	Proven IA diagnosis requires a combination of host factors, clinical evidence, and microbiological evidence. Detection of anti-*Aspergillus* antibodies is not useful due to impaired production; other biomarkers are recommended. Microscopy, culture, and galactomannan (GM) detection in BAL samples are recommended; serum GM has low sensitivity. Radiological findings are often atypical. Approximately 50% of patients may present with *Aspergillus* tracheobronchitis. CAPA-specific considerations: BAL microscopy, culture, and GM detection are recommended when possible. Only ~20% of CAPA patients have positive serum GM; a negative result does not exclude CAPA. Combining BAL culture, GM, and PCR may increase diagnostic specificity. Radiological findings are often atypical.

## Data Availability

No new data were created or analyzed in this study. Data sharing is not applicable to this article.
